# Non-linear canonical correlation for joint analysis of MEG signals from two subjects

**DOI:** 10.3389/fnins.2013.00107

**Published:** 2013-06-14

**Authors:** Cristina Campi, Lauri Parkkonen, Riitta Hari, Aapo Hyvärinen

**Affiliations:** ^1^Department of Computer Science/HIIT, University of HelsinkiHelsinki, Finland; ^2^CNR-SPINGenova, Italy; ^3^Department of Biomedical Engineering and Computational Science, Aalto UniversityEspoo, Finland; ^4^Brain Research Unit and MEG Core, O.V. Lounasmaa Laboratory, Aalto UniversityEspoo, Finland; ^5^Department of Mathematics and Statistics, University of HelsinkiHelsinki, Finland

**Keywords:** canonical correlation anaysis (CCA), non-linear CCA, magnetoencephalography (MEG), social interaction, brain signal processing

## Abstract

Traditional stimulus-based analysis methods of magnetoencephalography (MEG) data are often dissatisfactory when applied to naturalistic experiments where two or more subjects are measured either simultaneously or sequentially. To uncover the commonalities in the brain activity of the two subjects, we propose a method that searches for linear transformations that output maximally correlated signals between the two brains. Our method is based on canonical correlation analysis (CCA), which provides linear transformations, one for each subject, such that the temporal correlation between the transformed MEG signals is maximized. Here, we present a non-linear version of CCA which measures the correlation of energies and allows for a variable delay between the time series to accommodate, e.g., leader–follower changes. We test the method with simulations and with MEG data from subjects who received the same naturalistic stimulus sequence. The method may help analyse future experiments where the two subjects are measured simultaneously while engaged in social interaction.

## 1. Introduction

Magnetoencephalography (MEG) is a powerful functional neuroimaging method with a millisecond-scale temporal resolution (Hämäläinen et al., 1993). MEG is based on a non-invasive measurement of the extracranial magnetic field associated with neural currents in the brain. The aim of MEG experiments is to uncover the dynamical behavior of neuronal populations activated by a stimulus or a task.

This paper introduces a novel method for the analysis of MEG signals measured from two brains. The data can come from two different scenarios. In the simpler case, we have measurements of brain activity elicited by the same naturalistic stimulation. In the second case, which requires unconventional instrumentation, we consider data simultaneously measured from two subjects during social interaction, here referred to as two-person data (Baess et al., 2012). In general, such analysis would enable addressing neuroscientific questions on how we infer other person's intentions and how we develop mutual understanding by interaction with other people (Hari and Kujala, 2009; Hasson et al., 2012). In both cases, the goal is to identify near-simultaneous activations in the two brains (Hasson et al., 2004). The study of concurrent electrophysiological activity in interacting subjects has been investigated previously by electroencephalography (EEG), by recording from two persons simultaneously (“EEG hyperscanning”) (Babiloni et al., 2007; Dumas et al., 2010).

In the case of two-person data, we anticipate a small, fluctuating time delay between the activations in the two brains. Such lags are neurophysiologically interesting; they may signify successful predictions on other person's actions as well as leadership in the dyadic interaction. Obviously, during interaction the leader and follower can switch their roles, and the analysis method should be able to take this into account by allowing the delay to vary.

In general, searching for neural sources of unaveraged MEG data is very difficult because of the low signal-to-noise ratio (SNR). Moreover, common activations are unlikely tightly phase locked. Therefore, it is more reasonable to look for correlations of energies (powers) of the activations, rather than the source time series themselves, for identifying common sources.

## 2. Methods

### 2.1. Finding maximal lagged energy correlations

Let us denote the data measured from *i*-th subject, *i* = 1, 2, of the dyad, at the time point *t* as *b*_*i*_(*t*) = (*b*^1^_*i*_(*t*),…, *b*^*C*^_*i*_(*t*)) where *C* is the number of channels of the MEG sensor array. We estimate a source producing the magnetic field *b*(*t*) as the product *w*^T^*b*(*t*) where *w* is a spatial filter in the sensor space, i.e., *w* = (*w*^1^,… *w*^*C*^). We can straightforwardly write the energies of the sources producing the signals *b*_*i*_(*t*) in the following way:

(1) $$e_{i}(t)={\left(w_{i}^{\text{T}}b_{i}(t)\right)}^{2}={\left(\sum_{j\;=\;1}^{C}w_{i}^{j}b_{i}^{j}(t)\right)}^{2}.$$

A smoothed version of the energies can be obtained as a convolution with a temporal filter *h*. We chose *h* to be a Gaussian with a small standard deviation, based on the Fourier spectrum of the data, such that in the frequency domain it was large enough to cover the bulk of the spectral content of the data. In our framework, energy smoothing is enabled by a special signal representation which we introduce below. Thus, we first describe our method without smoothing and introduce it later.

The aim of this analysis is to find the spatial filters that maximize the correlation of the energies of the measurements from two subjects. We have to take into account that the best correlation can occur with some lag τ, which further depends on *t*. This parameter has to be estimated from the data, and the τ time-dependence introduces further complications into the analysis.

Thus, for estimating the spatial filters *w*_1_ and *w*_2_ and the lags τ(*k*), *k* = 1,… *K*, giving maximally correlated sources, we have to solve:

(2) $$\begin{array}{l} \underset{w_{1},w_{2},\tau }{\max}[\text{corr}(e_{1}(t),e_{2}(t-\tau (t)))]\\ \;=\underset{w_{1},w_{2},\tau }{\max}\left[\frac{cov[{\left(w_{1}^{\text{T}}b_{1}(t)\right)}^{2},{\left(w_{2}^{\text{T}}b_{2}(t-\tau (t))\right)}^{2}]}{\sigma ({\left(w_{1}^{\text{T}}b_{1}(t)\right)}^{2})\sigma ({\left(w_{2}^{\text{T}}b_{2}(t)\right)}^{2})}\right] \end{array}$$

where 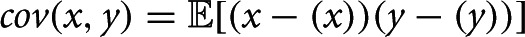 is the covariance, 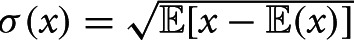 is the standard deviation and 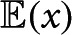 is the expected value of *x*.

A general method for finding maximally-correlated features in datasets is the Canonical Correlation Analysis (CCA) (Hotelling, 1936), which has recently been applied also to MEG measurements (Soto et al., 2010). Given two data sets, CCA computes two linear transformations such that once they are applied to the original data, the correlation between the transformed data sets is maximized. This method cannot be applied straightforwardly on Equation (2) for finding sources with maximally correlated energies since in this case the relationship between the energies and the spatial filters is quadratic and thus non-linear.

We implemented a Non-Linear CCA (NLCCA) in the following way: we expanded the energies *e*_*i*_ by considering at each time point *t* the space of all the binomials *B*^*jk*^_*i*_(*t*) = *b*^*j*^_*i*_(*t*)*b*^*k*^_*i*_(*t*), where both *j* and *k* go from 1 to *P*, obtained by multiplying all possible pairs of the *P* components of the data *b*_*i*_ after the Principal Component Analysis (PCA) pre-processing prior to CCA. PCA selects a fixed number of components explaning a certain amount of the variance in the data. The dimension of the data is hence reduced from *C*, the number of channels, to *P*, the number of components explaining more than 90% of the variance (*P* << *C*). For a fixed *t*, *B*_*i*_ is a vector with dimension *P*^2^ and we can associate it to a spatial filter with same length *W*_*i*_ = (*W*^1^_*i*_,…, *W*^*P*^2^^), obtained by expanding the vector *w*_*i*_ as *W*^*jk*^_*i*_(*t*) = *w*^*j*^_*i*_ (*t*) *w*^*k*^_*i*_(*t*).

In the space of binomials, the problem of finding *W*_1_, *W*_2_ which solve the problem in Equation (2) is equivalent to

(3) $$\underset{W_{1},W_{2}}{\max}\left[\frac{cov[W_{1}^{T}B_{1}(t),W_{2}^{T}B_{2}(t)]}{\sigma ((W_{1}^{T}B_{1}(t))\sigma (W_{2}^{T}B_{2}(t))}\right]$$

which is a linear problem. In this and the following equations the dependency of the maximization on the parameter τ was omitted for the sake of simplicity.

Here, we also see how smoothing of the energies is possible by a direct application of a temporal filter to the expanded data *B*_*i*_. In fact, we can simply smooth *B*_*i*_ by a linear filter because in a linear problem, the order of spatial and temporal filtering is irrelevant. In the following, we thus consider the smoothed version of the energies by simply assuming that *B*_*i*_ have been smoothened by the filter *h* described above.

For computational efficiency, we center and whiten *B*_*i*_. The covariance matrices *C*_*i*_ of the *B*_*i*_ are identity matrices, and the constraint on the norm of *W*^T^_*i*_ *C*_*i*_*W*_*i*_ is reduced to a constraint on the norm of *W*_*i*_. We also constrain *W*_*i*_ to unit Frobenius norm, which does not reduce generality since scaling does not affect the correlation coefficient. Then, Equation (3) can be written as: 
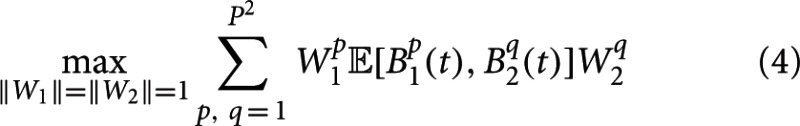
 where *p*, *q* are multi-indices such that *p*, *q* = (*P* − 1)^*^ *j* + *k*, and *j*, *k* = 1,…, *P*. This equation can be written for the original filters *w*_1_ and *w*_2_ as

(5) $$\underset{\|w_{1}\|=\|w_{2}\|=1}{\max}F(w_{1},w_{2})$$

where 
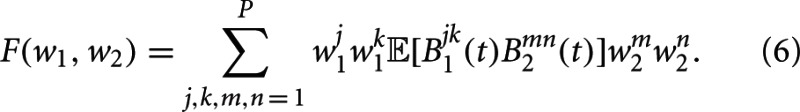


To perform the actual maximization, we first compute the derivative of this objective function with respect to the index *j*: 
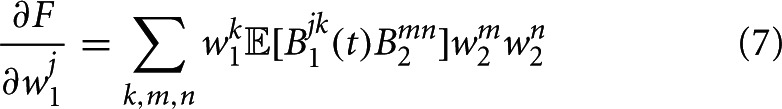
 and use it for iteratively updating the components as follows:

(8) $$w_{1}^{j}\leftarrow \frac{\partial F}{\partial w_{1}^{j}}$$

and similarly for the filter *w*_2_. This approach can be considered a variant of the power method for computing the dominant eigenvectors of a matrix, and it is essentially the same as a gradient method with an infinite step size. The infinite step size is not a problem due to the unit-norm constraint on the *w*_*i*_; this constraint is implemented by normalizing *w*_*i*_ after every iteration of Equation (9):

(9) $$w_{i}\leftarrow \frac{w_{i}}{\left\|w_{i}\right\|}.$$

For estimating τ, we maximize the objective function with respect to τ, in alternation with the maximization with respect to the spatial filters. We start by splitting the datasets in to a sequence of *K* windows and estimate, for each window, the τ giving the best correlation. The lags τ are restricted to a set of discrete values given *a priori* and in our case covering the interval of possible lags between the responses in the two brains. We also assume some continuity over adjacent windows: once τ(*k*) is found, the values tested for τ(*k* + 1) have to be close to τ(*k*) where *k* is the index over the windows, *i.e.*, they can still be τ(*k*) or assume one of the four values of the discrete τ that are closer to τ(*k*). If τ(*k*) is one of the extrema of the interval of possible lags, the values τ(*k* + 1) can assume are restricted to τ(*k*) and its two nearest values falling in the interval. In two-person data, the sign of τ gives the “direction” of the interaction, *i.e.*, the lag estimate in every window indicates which subject is the “leader” of the interaction.

Once the pair of filters (*w*_1_, *w*_2_) and the associated lag have been estimated, the data are projected on the space orthogonal to those filters to eliminate the part of the data represented by the estimated components. The method is then recursively applied to the projected data for estimating another pair of filters until all the *P* components are exhausted. This recursive application of the method is required to explore the whole space where sources are placed. No assumption on the orthogonality of the sources themselves is made but rather the orthogonality of the spatial filter is introduced by our algorithm, as a tool for investigating the data. Component pairs with high correlations will presumably represent the interesting content of the data, in this case the common activations, while the pairs with low correlations are likely to be associated with noise.

### 2.2. Synthetic data

The method described in the previous section was applied both to synthetic and real MEG measurements. The synthetic data were designed to test in which conditions our algorithm can discriminate correlated and anti-correlated sources in two datasets from a two-person MEG experiment.

We simulated two sources in both of the two brains, one in the occipital lobe and the other in the left parietal lobe close to the midline. No delays were introduced in this simulation. For Subject 1, we simulated strong rhythmic activity in the occipital lobe, occuring at the same time as a weaker activity in the parietal lobe. After a period of no activity, the activations were “flipped”; strong rhythmic activity appeared in the parietal source and a weak one in the occipital source and so on. For Subject 2, timing of the envelopes of the oscillations was the same as for Subject 1, but the strengths in occipital vs. parietal lobe were reversed.

**Figure 1:**
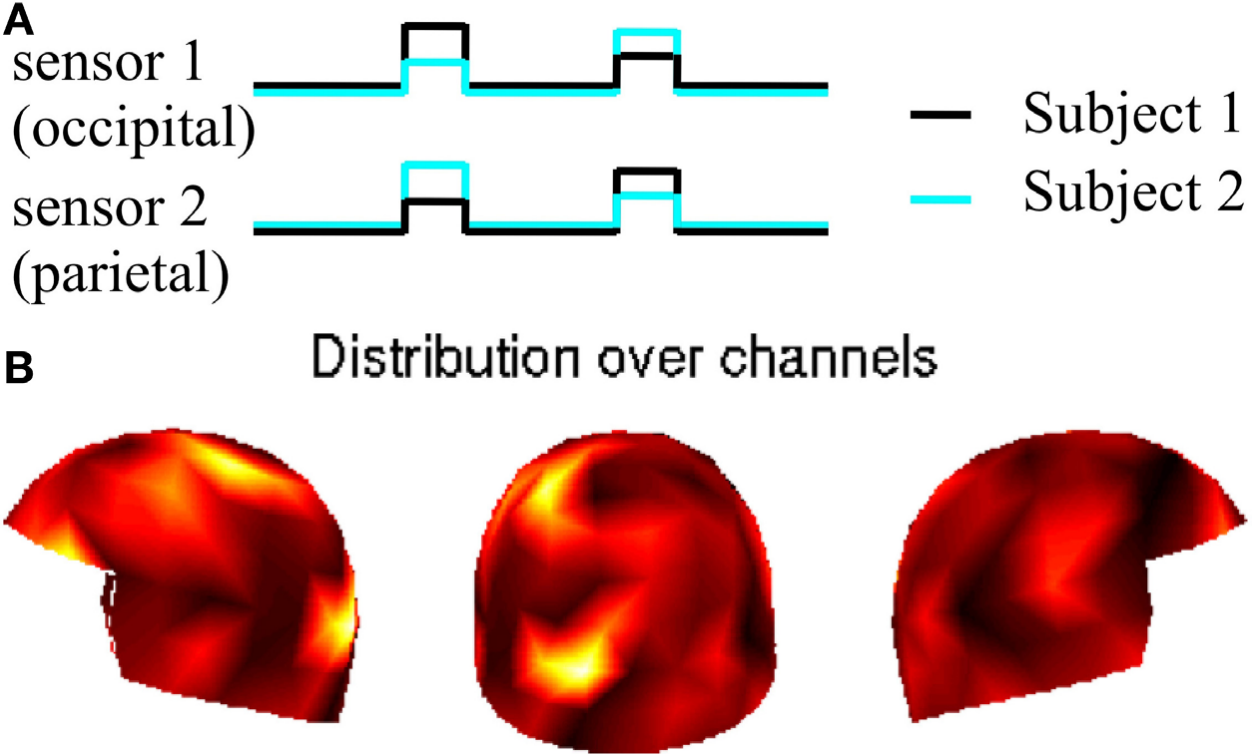
**Experimental setting with synthetic data**. Panel **(A)**: envelopes and source locations of the simulated activations for Subjects 1 and 2. Panel **(B)**: distribution of the resulting planar gradients over the sensor helmet of one subject; the helmet is viewed from left, back, and right.

These sources produced the strongest signals in occipital and parietal areas slightly leftwards from the midline; see Figure 1. The total number of data points was 36,000, and the peaks of the rhythmic signals were separated by 300 data points.

Once the source locations and envelopes were chosen, the two resulting magnetic field time courses were simulated using lead field matrices. These matrices were computed using a boundary-element method (BEM) with a single compartment (Hämäläinen and Sarvas, 1989) and a sensor array formed by 204 planar gradiometers and 102 magnetometers. The inner skull surface needed in this approach was created by the FreeSurfer software (Dale et al., 1999) the sentence from a structural MRI of a healthy adult. In the following, only the data coming from the gradiometers were taken into account. Brain noise from a real MEG experiment, where the subject was silently resting, was added to the data so that at the peak of the activation the SNR was 2.4 dB. A PCA was applied to both data sets and 10 components explaining more than 90% of the variance were selected. These 10 components for the two subjects were used as the input for our algorithm.

We set the weak source to be either 50 or 30% of the strength of the strong source.

### 2.3. Naturalistic stimulation data

Next, we used previously recorded real MEG data (Ramkumar et al., 2012) obtained from 11 subjects (after written informed consent) who had received the same sequence of randomly-alternating blocks of visual, auditory, and tactile stimulation interspered with rest periods for a total duration of about 8 min. The recordings had prior approval by the Ethics Committee of the Helsinki and Uusimaa Hospital District. MEG signals were acquired with a 306-channel (204 planar gradiometers and 102 magnetometers) Elekta Neuromag MEG system (Elekta Oy, Helsinki, Finland), filtered to 0–200 Hz and digitized at 600 Hz. Only the data measured by the 204 planar gradiometers were used in the analysis. To investigate the capability of our method to estimate lags τ, artificial lags were introduced.

The data were used in two different experiments:

1. First, all the possible couplings of the 11 subjects were considered for creating 55 pairs. In all these pairs, the data of one subject were artificially delayed with respect to the other subject by 0, 100, 250, 500, or 1000 ms.
2. Second, the subjects were divided into two groups of five subjects and the data were concatenated in time to build two long datasets.

The rationale of the first setting was to test the ability of our method to detect lags between expected correlated activations in the pairs of measurements. The delay in the data was constant for the whole recording, but this does not reduce the generality of the experiment too much since the estimated lag was not constrained to be constant.

The second setting was used to test whether increasing the amount of data would improve the accuracy of estimating the spatial filters.

In both cases, all the data were filtered to the frequency band of 8–30 Hz and dowsampled to 150 Hz. Altogether 30 components, explaining more than 90% of the variance, were extracted by PCA for all the pairs with the five different artificially-introduced delays and for the concatenated datasets.

For the first setting, we set the interval of admissible lags to [−750, 750] ms for the case of 0, 100, 250, and 500 ms delays, while for the 1000-ms delay the interval of admissible lags was [−1500, 1500], with a step 100 ms. The length of the window used for estimating the spatial filters and the lags was 10 s in all cases.

## 3. Results

### 3.1. Synthetic data

**Figure 2:**
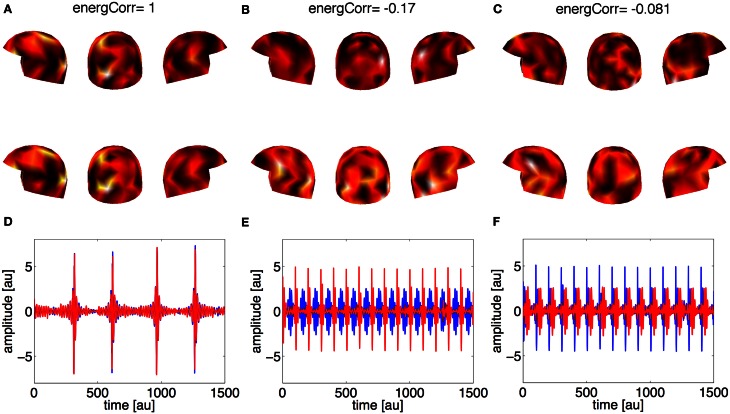
**Synthetic data, Case 1: the strength of the weak source is 50% of the strong source**. Panels **(A–C)** represent the first three estimated spatial filters over the sensors. On the top of each panel the correlation coefficient is reported. The upper row of helmets is for Subject 1, the lower row for Subject 2. Panels **(D–F)** contain waveforms (red line for Subject 1, blue line for Subject 2) associated to the three estimated spatial filters.

In the first case (the weak source 50% of the strong one), the method estimated just one relevant component, while the other components represented noise, see Figure 2. Correlation coefficients permitted to distinguish between the components representing activations and the components representing noise: almost 1 for the former, and clearly lower (around 0.15) for the latter ones. In this case the method cannot separate the two sources in the brains of the two subjects, presumably because of the strong correlation of the sources meaning that all the activations were already included in the first component.

**Figure 3:**
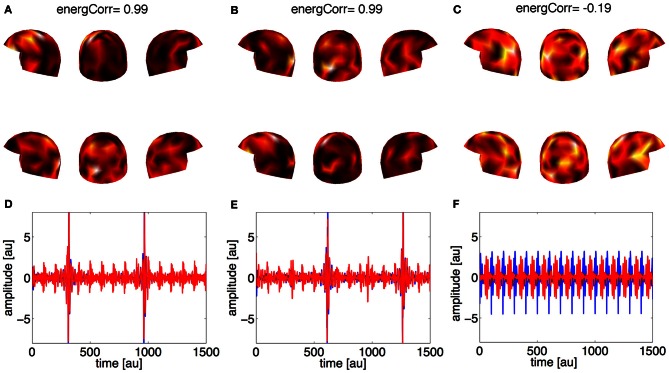
**Synthetic data, Case 2: the strength of the weak source is 30% of the strong source**. Panels **(A–C)** represent the first three estimated spatial filters over the sensors. On the top of each panel the correlation coefficient is reported. The upper row of helmets is for Subject 1, the lower row for Subject 2. Panels **(D–F)** contain the waveforms (red line for Subject 1, blue line for Subject 2) associated to the three estimated spatial filters.

In the second case (the weak source 30% of the strong one), the method was further able to properly separate the locations of the coupled sources: the first two estimated components (Figures 3A,B) represent the correlated activities in the two subjects, while the third component and the following ones (not shown) represent noise. This distinction between signal and noise is again possible using the correlation coefficients. The second row of Figure 3 shows the waveforms associated with the estimated spatial filters: panels **(D)** and **(E)** show the representation of the simulated activities for both subjects, while in panel **(F)** the activities are no more coupled.

### 3.2. Real measured data

**Figure 4:**
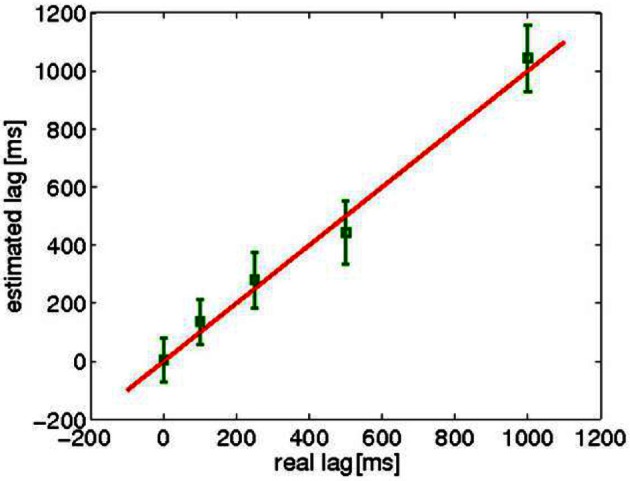
**The mean ± standard deviation, over all 55 pairs and all the time points of the estimated lags for the artificially delayed data**. The theoretical x = y line is also shown.

Figure 4 shows the averages of the estimated lags as a function of the artificially inserted delay. These values are averages over all 55 pairs and all time-windows. The correlation (*r* = 0.74, *p* < 0.05) indicates that the algorithm works well in the estimation of the lags for the artificially delayed datasets.

**Figure 5:**
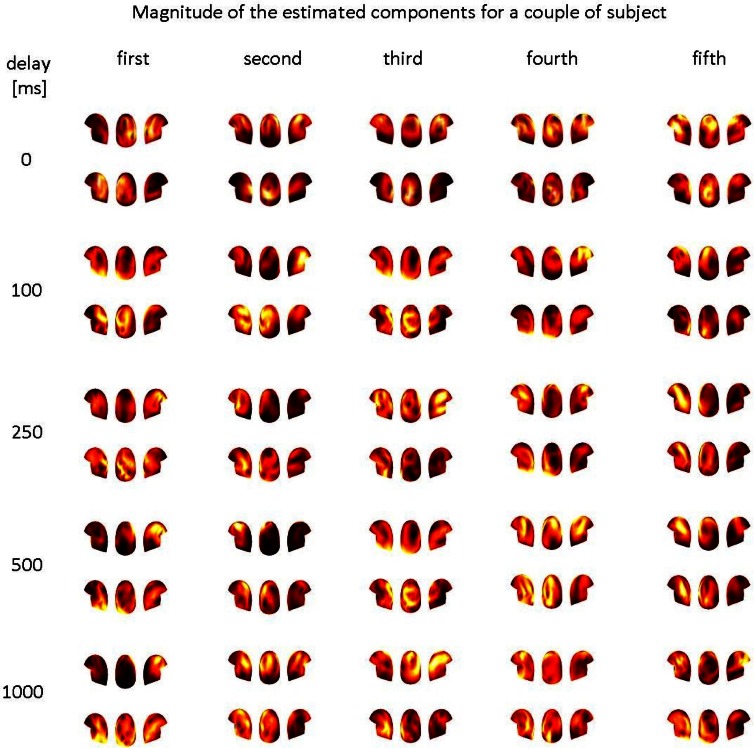
**The magnitudes of the five estimated spatial filters for one pair of subjects: each column represents a filter, each row represents the result for one of the five artificially inserted delays**. For all the five delays, the three views (left, back, right) of the helmet in the first row are for the first subject of the pair, while the three views in the second row are for the second subject.

However, the results of the estimated spatial filters were less promising. In Figure 5, one of the 55 pairs was selected and five of the estimated spatial filters for the 5 artificial delays are shown. The distributions differ from our expectations of similar spatial patterns in both subjects. It is not easy to see any systematic connection between the spatial distributions in the two brains.

**Figure 6:**
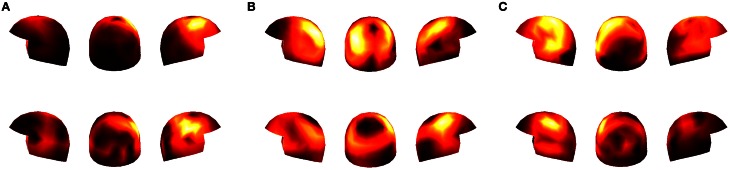
**Panels **(A–C)**: the distribution over the sensors of three spatial filters estimated by our method for the concatenated datasets**. The helmets in the first row represent concatenated data of Subjects 1, 2, 3, 4, and 5, and the second row concatenated data of Subjects 6, 7, 8, 9, and 10.

This negative result provided the motivation for considering data concatened over subjects. Concatenated datasets (with data from five subjects each) were thus analyzed to find out whether a larger amount of data would allow our algorithm to detect coupled activation between the subjects. In fact, as the first row of Figure 6 shows, our method now found similar spatial filters in the concatenated data.

## 4. Discussion

We proposed a data-driven method based on non-linear canonical correlation analysis for finding, on the basis of MEG recordings from two subjects, linear transformations of the data representing cerebral sources with maximally correlated energies, allowing a delay of 0–1500 ms. A simple algorithmic implementation was proposed by expanding the data to the space of all possible products of the channels. Application to synthetic and semi-real two-person data sets indicated that the method is promising.

The experiments with synthetic data pointed out that the method can recover the original, independent sources in the data provided that the relative differences between the amplitudes of the sources are large enough. Otherwise, this method may include all the sources in a single component, as a kind of a larger network. An important question is whether and how the separation capability of the method could be improved. On the other hand, estimating such coarse large networks may also be useful in some applications.

In experiments with real, although articifically delayed MEG recordings, NLCCA estimated correctly the delays even in single subjects. In contrast, the estimated spatial filters did not always represent reliable coupled activations for single subjects. The method seems to require more data for reliable estimation of spatial filters as was seen in the long datasets created by concatenating measurements from different subjects, for which the estimated spatial filters were similar enough in the two data sets. The concatenation, although useful for the estimation of the spatial filter, precludes real-time analysis of the data. A statistical optimization of our energy correlation measures to enable reliable estimation in single subjects is an important topic for future research. Essentially the same problem of finding maximally correlated energies was considered by Gutmann and Hyvärinen (2011), but by using a rather different method. When applied on the same naturalistic stimulation data, their method did not seem to find the correct coupling (results not shown), presumably because the correlation in noisy data was not strengthened by temporal smoothing. Another difference with respect to the method of Gutmann and Hyvärinen (2011), is the estimation of sources with maximally correlated energies for some lag τ.

The search of sources with maximally correlated energies could be carried out on the source time series estimated by some inversion method but in this work we preferred to investigate the possibility to operate directly on the MEG data, without introducing the inversion step.

Further methodological developments are required for the analysis of data measured during real two-person interaction. The delay parameter τ assumes a crucial role in such analysis, and its estimation, due to its time dependency, induces a great computational cost. Moreover, it would be important to estimate τ, and consequently the often unpredictable changes in the leadership of the interaction, with a good temporal accuracy which would require shorter time windows than what we had in the current work. The consequent problems in NLCCA estimation form a further motivation for future research.

### Conflict of interest statement

The authors declare that the research was conducted in the absence of any commercial or financial relationships that could be construed as a potential conflict of interest.
